# The Differential Impact of Three Different Anesthetics on Large-Scale Neuronal Activity Measured Using Voltage-Sensitive Dye Imaging in Rat Brain Slices

**DOI:** 10.1213/ANE.0000000000007616

**Published:** 2025-06-10

**Authors:** Ana Ghenciulescu, Jaideep J. Pandit, Ian M. Devonshire, Susan A. Greenfield

**Affiliations:** From the 1 Nuffield Department of Anaesthetics, Oxford University Hospitals NHS Foundation Trust, Oxford, UK; 2 Nuffield Department of Clinical Neurosciences, University of Oxford, Oxford, UK; 3 Nottingham University Medical School, Queen’s Medical Centre, Nottingham, UK; 4 Neuro-Bio Ltd, Culham Science Centre, Abingdon, UK.

In the study of neural mechanisms concerning hypnotic actions of anesthetics, a mid-level of brain operations has been identified as particularly appropriate: “neuronal assemblies” are extensive coalitions of neurons transiently activated over a subsecond time frame, functioning between the micro-level of cellular operations and the “top down” level of anatomically defined brain regions. Neuronal assemblies have been previously characterized empirically, both in in vivo (rat)^1^ and in brain slices ex vitro.^2^ Generation of a neuronal assembly is unlikely the result of synaptic transmission alone but involves 2 further signaling systems: “volume” transmission (passive diffusion of diverse bioactive transmitters, hormones and modulators), and electronic spread via gap junctions.^3^

Assemblies are studied by optical imaging using voltage-sensitive dye imaging (VSDI)^4^ that reflect cellular depolarization graded over several millimeters of brain tissue following an electrical stimulus.^4–6^ As such, they have provided insight into a range of scenarios, including urethane anesthesia in rat barrel cortex^7^ and differentiation of anesthetics (thiopental and propofol) from analgesics (morphine and gabapentin) in hippocampal slices.^2^ Kratzer et al reported using VSDI that propofol and sevoflurane differentially modulate cortical depolarization of the ventrobasal thalamus.^8^

We wished to adopt the approach and rationale of these earlier studies to investigate if other general anesthetics might modulate assemblies as measured in the key brain regions plausibly involved in mechanisms of consciousness: in addition to coronal sections, studying para-sagittal slices containing active thalamocortical projections. Since there is increasing evidence that different agents act by different fundamental mechanisms, in part through receptor selectivity^9^ we also hypothesized that VSDI response patterns would not be identical across agent classes.

## METHODS

The methods have been previously described,^1–5,7^ and supplementary material provides analytical details (Supplemental Digital Content, Figures S1–S8, http://links.lww.com/AA/F382 explaining the metrics used in final analysis). Briefly, brain sections of 14-day male Wistar rats (all procedures conformed to ARRIVE guidelines) were subjected to the VSDI technique, with 3 regions of interest: agranular insular frontal cortex (AIC), primary somatosensory barrel field (S1BF), and a section with the thalamocortical projection intact (TC). VSDI can generate 3 quantitative parameters (see Supplemental Digital Content, Figure S5, http://links.lww.com/AA/F382): (a) summed fluorescence (a measure of the overall magnitude of brain response/activity); (b) lateral spread (of evoked responses, in mm); (c) time course (of activity, measured in ms).

We studied pentobarbital (a barbiturate), etomidate (an imidazole derivative), and ketamine (an arylcyclohexylamine derivative). Although the first 2 share action on GABA receptors, the full spectrum of activity across all their molecular targets appears unique for these agents.^10^

Experimental data are plotted for clarity in curve-fitted graphs as individual data points with curve-fitting, or as “superplots” (median, interquartile range and individual data points).^11^ Derived parameters from curve fitting are described by mean (SD). Concentration–response relationships were plotted, with lines of best fit constructed using nonlinear least squares regression, with models fit empirically to a simple ligand binding equation (Sigmaplot for Windows Vers 11, Grafiti LLC):


$$\begin{array}{l} Y=\frac{\left[B\max\right]\cdot \left[X\right]}{\left[Kd\right]+\left[X\right]} \end{array}$$

where Bmax is the estimated maximum effect of drug (normalized values; see Supplemental Digital Content, http://links.lww.com/AA/F382), [X] is the drug concentration (%), and Kd is the dissociation constant (%). Statistical comparisons were made between the mean (SD) values of Kd and Bmax using factorial analysis of variance, with the factor “agent” having 3 levels, one for each agent, with statistical significance set at *P* < .05. Although we made no assumptions of normality in our data, we confined any statistical testing only to the parameters of the fitted dose-response curve derived from the data, such as Kd and Bmax, as described above, and not to the data themselves. The regression algorithms for the fitted dose-response curves minimize the difference between the model’s predictions and the observed data points. Thus, each parameter estimate is a function of all the individual data points, with no assumptions made about underlying data distribution. Moreover, the central limit theorem states that the distribution of the sample mean (or in this case, the parameter estimate derived from the sample) will tend towards a normal distribution as the total number of data points increases, regardless of the underlying distribution. In the context of dose-response curves, each parameter estimate can be thought of as a summary statistic derived from the collection of response measurements at different doses, generating outputs as a mean (standard deviation [SD]). Analysis of variance (ANOVA) is therefore appropriate, especially given its power in respect of testing for interactions and its robustness to departures from normality even when individual samples are as small as n = 5.^12^

The main hypothesis being tested was whether, for the primary end-point of summed fluorescence, the concentration-response curves across the agents were significantly different, with no physiological implications being ascribed to any particular pattern of response, or to any differences at any particular dose. In sample size, we were guided by the approach of Johnstone et al who used a minimum of 3 separate concentrations (plus the zero point) with a minimum of 3 repeats (in independent cells from different animals) at each concentration, to construct a suitable dose-response curve.^13^ We studied between 4-6 concentrations for the main end-point, and a minimum of 6 repeats (sections).

## RESULTS

**Figure 1.**
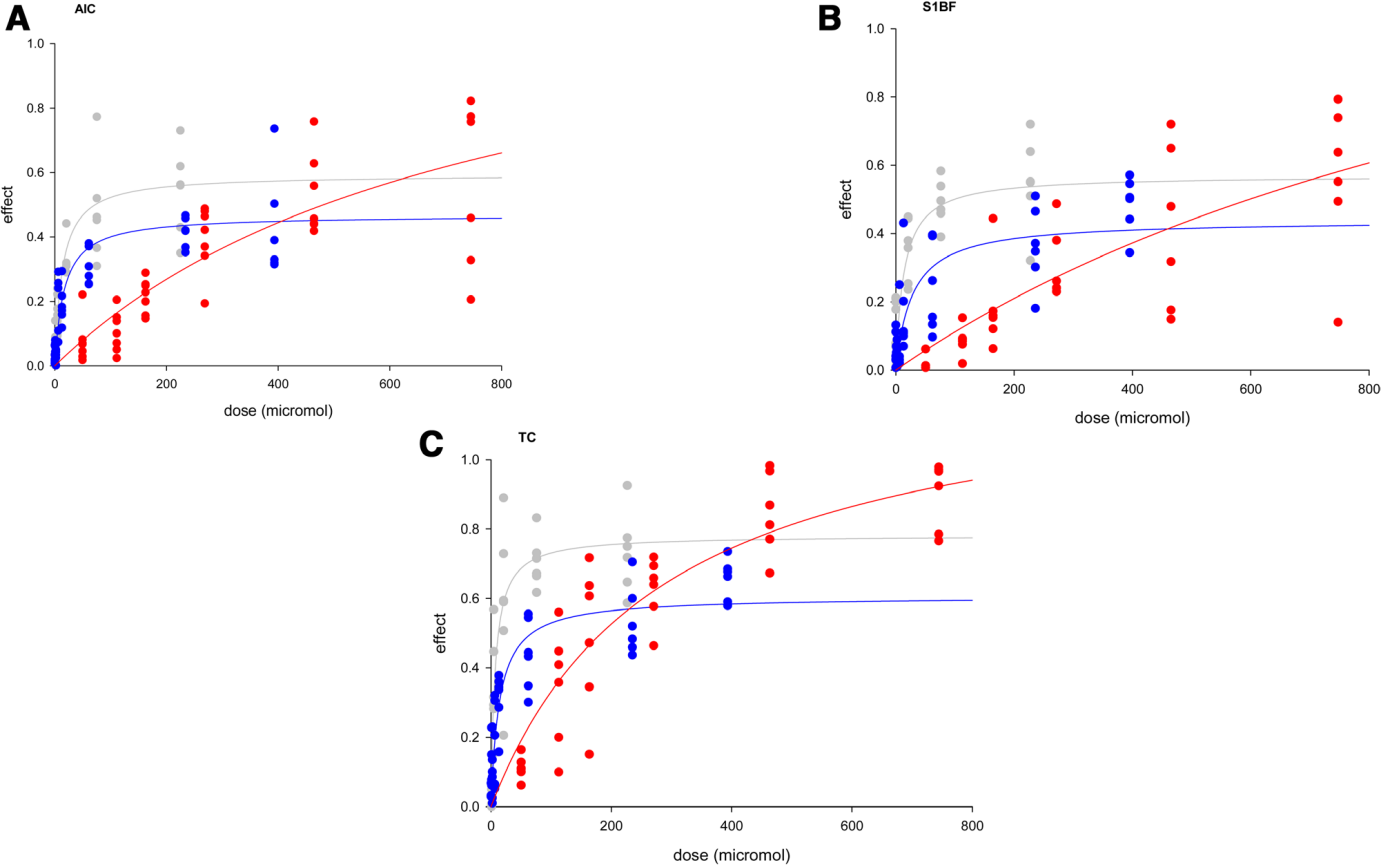
Summed fluorescence concentration–response relationships (normalized measure; see supplementary material for normalization procedure, http://links.lww.com/AA/F382) of etomidate (gray), ketamine (blue), and pentobarbital (red) on (A) AIC, (B) S1BF, and (C) TC. Individual data points are plotted. Calculated values for mean (SD) for Bmax and Kd are shown in Supplemental Digital Content, Table S1 (http://links.lww.com/AA/F382), along with statistical comparisons. The number of independent sections (different animals) were for AIC, ketamine: n = 6, etomidate: n = 6, pentobarbital: n = 7; for S1BF, ketamine: n = 6, etomidate: n = 6, pentobarbital: n = 6; for TC, ketamine: n = 6, etomidate: n = 6, pentobarbital: n = 6; with repeated measurements (by dose) taken in each section.

For summed fluorescence, all anesthetics influenced all 3 parameters of neuronal assemblies in the 3 brain regions, in a dose-related manner across clinically relevant concentrations, with a good fit to the single ligand-receptor binding model (Figure 1 and Supplemental Digital Content, Table S1, http://links.lww.com/AA/F382). Etomidate and ketamine generally exhibited saturation in all regions: etomidate showed greater efficacy but ketamine showed greater efficacy for lateral spread. Pentobarbital did not appear to show saturation by the model fit and had the lowest potency.

**Figure 2.**
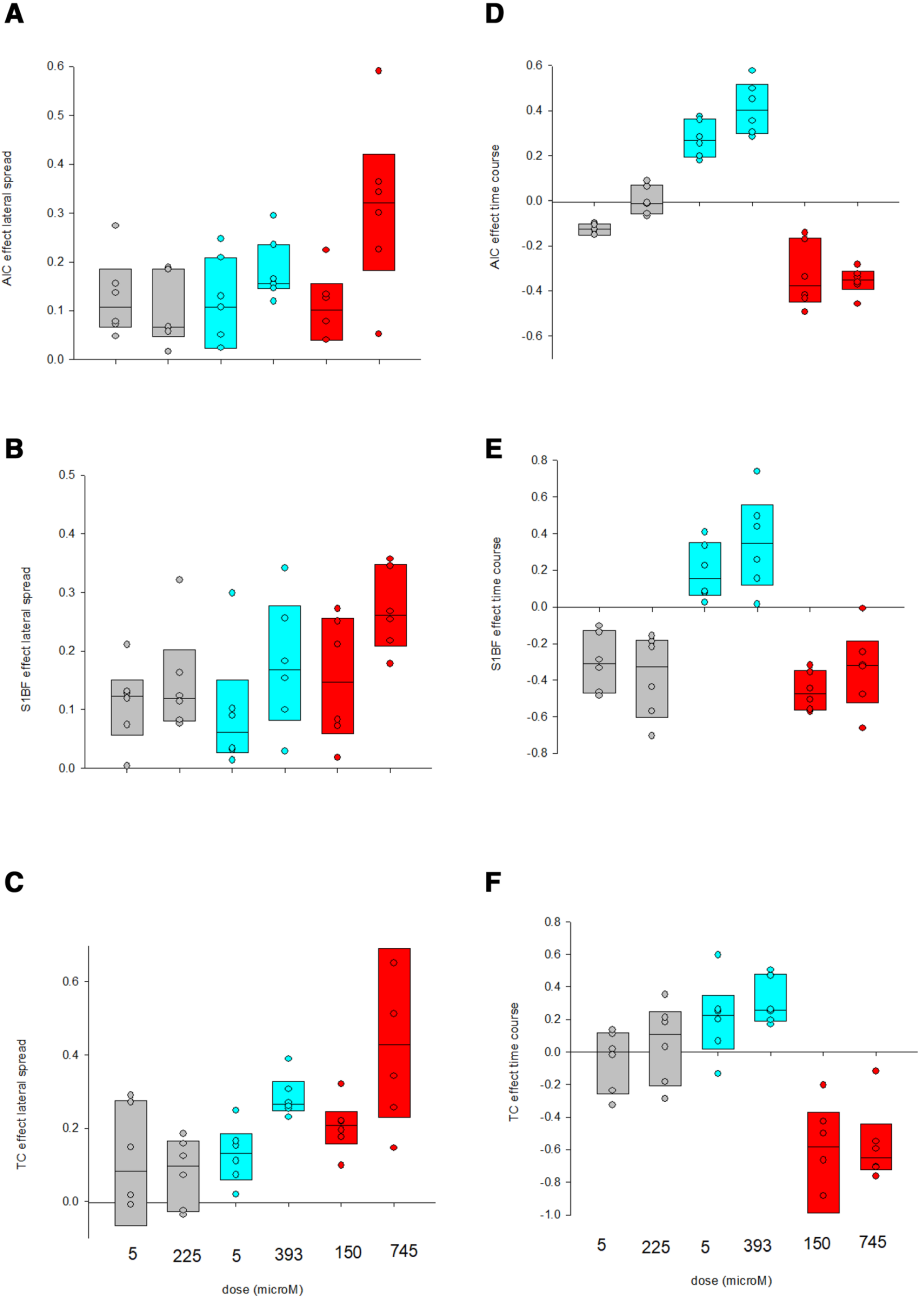
Superplots (boxplots where horizontal line is the median and outer edges are the 25th to 75th centiles, with individual data points shown) vs drug concentration–response for lateral spread (A–C) and time course of activity (D–F) for each of the 3 brain sections: AIC (A and D); S1BF (B and E); and TC (C and F). For statistical comparison, see Supplemental Digital Content, Table S2 (http://links.lww.com/AA/F382). The number of independent sections was 6 for each drug, with repeats across the doses stated.

The results for lateral spread and time course were more complex and could not be readily fit to ligand binding curves (Supplemental Digital Content, Figure S9, http://links.lww.com/AA/F382). We plotted these as superplots to indicate the median and spread of data by drug concentration (Figure 2), and present factorial analysis of variance results for conditions of “drug,” “dose” and the interactive term (drug x dose) in Supplemental Digital Content, Table S2, http://links.lww.com/AA/F382. The results reflect agent-specific differences in effect on these parameters.

## DISCUSSION

The main results of this study are as follows: first, anesthetics influence neuronal assemblies in brain regions of interest in ways that are consistent with a hypnotic effect, across clinically relevant concentrations. Second, at the level of synaptic transmission as demonstrated by assembly function, each of the 3 anesthetics can be differentiated from the other 2.

While a classical drug-receptor binding model can fit the data well for certain dimensions of the assembly (summed fluorescence and, notwithstanding limited data points, also lateral spread; Figure 1 and Supplemental Digital Content, Figure S9A–C, http://links.lww.com/AA/F382), the pattern of responses for time course are more difficult to explain, especially where these result in responses in different directions across the agents (Figure 2D–F and Figure S9D–F). The drug-receptor binding model is overly simplistic; that is, a good model fit does not imply that the drugs are acting on a single binding site, when we know assemblies arise from numerous synaptic interactions. Similarly, the time course results suggest more complex nonlinear relationships, indicating that additional processes other than familiar synaptic signaling, including electrotonic spread through gap junctions and nonspecific “volume” transmission may be involved.

We used a very wide range of anesthetic concentrations that encompassed the clinically relevant range but also went beyond, to obtain a full “dose-response” relationship. Nevertheless, the effects of pentobarbital did not always appear to saturate even at the highest concentrations. We used agents that are decidedly anesthetic, and different from agents previously reported using VSDI, such as thiopental, propofol, and sevoflurane.

Ketamine (for effect on summed fluorescence; Figure 1) and etomidate (for lateral spread; Supplemental Digital Content, Figure S9A–C, http://links.lww.com/AA/F382) appear to behave as “partial agonists” when data are fit to a ligand binding model. This raises the possibility that mixtures of these agents could exhibit competitive, inhibitory behavior, as has been shown for other agents in other experimental constructs.^14^

In summary, the study of assemblies using VSDI can offer insights into differences between agents at meso-level, as the basis for their established differential effects on macro-measures such as electro-encephalography (EEG) signaling.

## ACKNOWLEDGMENTS

We are grateful to Scott Badin for initial data collection and analysis.

## DISCLOSURES

**Conflicts of Interest:** S. A. Greenfield is CEO of Neuro-Bio Ltd and holds shares in the company; and as Baroness Greenfield is member of the UK House of Lords (UK legislature). J. J. Pandit is Editor-in-Chief, Anesthesia & Analgesia, and was not involved in the handling of this paper. No other authors declared Conflicts of Interest. **Funding:** This work was supported in part by a European Society of Anesthesiology Project Grant “Characterisation of spatio-temporal heterogeneity of anaesthetic action using voltage sensitive dye imaging and electrophysiology” to J.J.P. and S.A.G. **This manuscript was handled by:** Peter A. Goldstein, MD.

## Supplementary Material



## References

[1] Devonshire IM Dommett EJ Grandy TH Halliday AC Greenfield SA. Environmental enrichment differentially modifies specific components of sensory-evoked activity in rat barrel cortex as revealed by simultaneous electrophysiological recordings and optical imaging in vivo. Neuroscience. 2010;170:662–669. 20654700 10.1016/j.neuroscience.2010.07.029

[2] Collins TF Mann EO Hill MR Dommett EJ Greenfield SA. Dynamics of neuronal assemblies are modulated by anaesthetics but not analgesics. Eur J Anaesthesiol. 2007;24:609–614. 17261214 10.1017/S0265021506002390

[3] Badin AS Fermani F Greenfield SA. The features and functions of neuronal assemblies: Possible dependency on mechanisms beyond synaptic transmission. Front Neural Circuits. 2017;10:114. 28119576 10.3389/fncir.2016.00114 PMC5223595

[4] Grandy TH Greenfield SA Devonshire IM. An evaluation of in vivo voltage-sensitive dyes: Pharmacological side effects and signal-to-noise ratios after effective removal of brain-pulsation artifacts. J Neurophysiol. 2012;108:2931–2945. 22972958 10.1152/jn.00512.2011

[5] Mann EO Suckling JM Hajos N Greenfield SA Paulsen O. Perisomatic feedback inhibition underlies cholinergically induced fast network oscillations in the rat hippocampus in vitro. Neuron. 2005;45:105–117. 15629706 10.1016/j.neuron.2004.12.016

[6] Newton TH Reimann MW Abdellah M Chevtchenko G Muller EB Markram H. In silico voltage-sensitive dye imaging reveals the emergent dynamics of cortical populations. Nat Commun. 2021;12:3630. 34131136 10.1038/s41467-021-23901-7 PMC8206372

[7] Devonshire IM Grandy TH Dommett EJ Greenfield SA. Effects of urethane anaesthesia on sensory processing in the rat barrel cortex revealed by combined optical imaging and electrophysiology. Eur J Neurosci. 2010;32:786–797. 20646050 10.1111/j.1460-9568.2010.07322.x

[8] Kratzer S Mattusch C Garcia PS. Propofol and sevoflurane differentially modulate cortical depolarization following electric stimulation of the ventrobasal thalamus. Front Comput Neurosci. 2017;11:109. 29321737 10.3389/fncom.2017.00109 PMC5732174

[9] Urban BW. Current assessment of targets and theories of anaesthesia. Br J Anaesth. 2002;89:167–183. 12173228 10.1093/bja/aef165

[10] Rudolph U Antkowiak B. Molecular and neuronal substrates for general anaesthetics. Nat Rev Neurosci. 2004;5:709–720. 15322529 10.1038/nrn1496

[11] Lord SJ Velle KB Mullins RD Fritz-Laylin LK. SuperPlots: Communicating reproducibility and variability in cell biology. J Cell Biol. 2020;219:e202001064. 32346721 10.1083/jcb.202001064 PMC7265319

[12] Blanca MJ Alarcón R Arnau J Bono R Bendayan R. Non-normal data: Is ANOVA still a valid option? Psicothema. 2017;29:552–557. 29048317 10.7334/psicothema2016.383

[13] Johnstone RH Bardenet R Gavaghan DJ Mirams GR. Hierarchical Bayesian inference for ion channel screening dose-response data. Wellcome Open Res. 2016;1:6. 27918599 10.12688/wellcomeopenres.9945.2 PMC5134333

[14] Pandit JJ Huskens N O’Donohoe PB Turner PJ Buckler KJ. Competitive interactions between halothane and isoflurane at the carotid body and TASK channels. Anesthesiology. 2020;133:1046–1059. 32826405 10.1097/ALN.0000000000003520

