# Supplementary material for: The Differential Impact of Three Different Anesthetics on Large-Scale Neuronal Activity Measured Using Voltage-Sensitive Dye Imaging in Rat Brain Slices
Source: Anesth Analg. 2025 Jun 10;142(1):181–5. doi: 10.1213/ANE.0000000000007616 (PMC12677332; doi:10.1213/ANE.0000000000007616)
Supplement: Supplementary file 1 [file ane-142-181-s001.pdf]

Online Supplement: Methods

Anesthetic agents

Anaesthetic drug solutions for pentobarbital and ketamine were diluted in saline before administration to appropriate micromolar concentrations. Etomidate was first dissolved in a small volume of DMSO, with final DMSO proportions never reaching more than 0.1% v/v in final preparation. Due to the broad range of estimated EC50s for the agents on their molecular targets (from 1-200 μM), a range of concentrations was employed to provide a full dose-response relationship for drug effects; 20-200 μM for etomidate, 20-400 μM for ketamine and 50-800 μM for pentobarbital. All chemicals, unless stated otherwise, were from Sigma-Aldrich Ltd. (UK).

Animals and tissue slice harvesting

Two-week-old Wistar rats (Charles River, UK) underwent terminal anesthesia using isoflurane followed by decapitation. The brain was submerged in ice-cold artificial cerebral spinal fluid (aCSF, in mmol: 124 NaCl, 22 NaHCO3, 10 glucose, 5 KCl, 2 CaCl2, 1.25 MgSO4, 1.25 NaH2PO4) for approximately 1 min. Coronal sections, 400 mm thick, were cut on a vibratome (Leica VT1000S) in ice cold oxygenated aCSF. Slices for two of the locations were obtained within the following regions in relation to bregma; AIC: between +3.70 to -2.20 mm; S1BF: between -1.60 and -3.14 mm. The TC slice was 50° in the sagittal plane and 10° in the coronal plane, to retain thalamocortical connections (Figure S1).

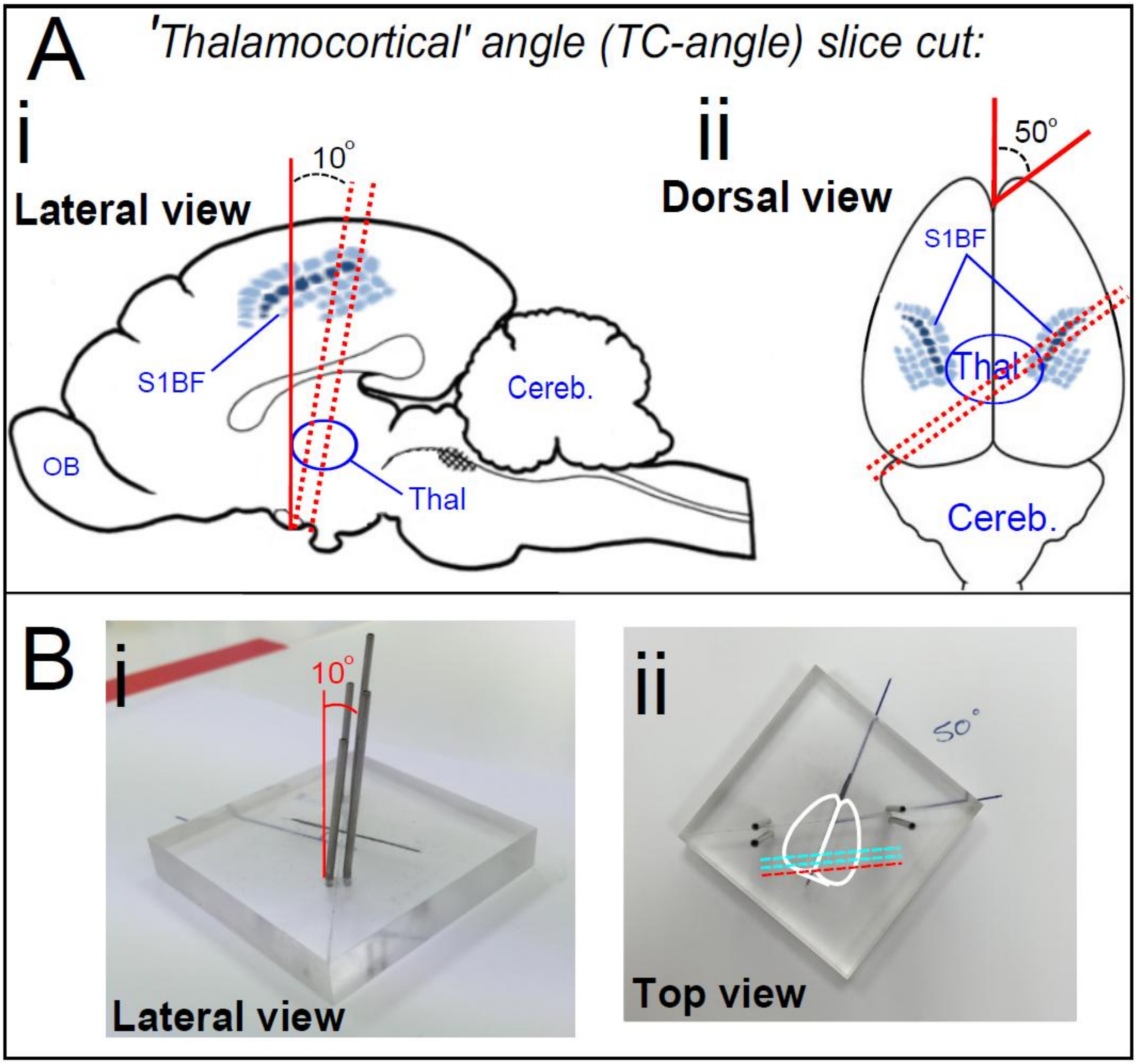

Figure S1. Slicing of thalamocortical angle cut. A. Schematic representation of rat brain showing the angles required to isolate functional thalamocortical slices. These were achieved using a small custom-made stage (B) to guide the blade and which achieved both the vertical 10° (i) and lateral 50° (ii) angles in a single cut, performed within seconds following removal of the brain from the skull, in a receptacle filled with ice-cold aCSF. Dotted lines in A and Bii represent the approximate location of active thalamocortical slices (in all cases, slices were 400 µm-thick).

Slice staining and imaging

Following slicing, sections were given 1 h to equilibrate at room temperature in a high humidity chamber with oxygenated aCSF before dye solution was applied for 25 min in a dark

box. Dye solution contained 4% 0.2 mmol Di-4-ANEPPS (Molecular Probes, Oregon, USA) in ethanol 2.7%, cremophor EL 0.13%, fetal bovine serum 48%, aCSF 24% and aCSF cellulose 24%. The dye was rinsed off by gentle agitation in aCSF and the sections were allowed to recover for 1 h in the humidity chamber with oxygenated aCSF. Once placed in the imaging chamber, a Minipuls 3 peristaltic pump (Gilson U.K., Luton, Bedfordshire, UK) delivered aCSF at a flow rate of 1.5 mL/min and maintained at 30 ± 1°C, monitored using a bath thermometer. A halogen lamp excited the dye through a 530 ± 10 nm filter and emission captured on a high-speed 10x10 mm, 100x100 pixel CMOS sensor (MiCam Ultima, BrainVision Inc., Japan) through a filter (>590 nm). The optics used resulted in spatial resolution of 40 μm for each pixel; sampling frequency was 1kHz.

Slice stimulation

Slice activity was evoked with a bipolar, concentric tungsten micro-electrode (CBARC75, FHC, USA) with impedance of 500 kΩ (measured at 1 kHz). The tip of the electrode consisted of a central pole (25 μm in diameter) made up of a platinum and iridium alloy, and inserted within a stainless steel tube providing the final diameter of the electrode tip at 125 μm. Electrodes were advanced 10-30 μm into each slice with the help of a white light stereoscope (4.5x magnification, Micro Instruments Ltd, England) and manual micromanipulator (Marzhauser Wetzlar, Germany). The AIC was stimulated directly in superficial layers (layers II/III) at 30 v for 0.1 ms. In S1BF slices, cortical activity was evoked by stimulating layer IV at 30 v for 0.1 ms. In TC slices, cortical activity was evoked by stimulating the ventroposterior medial nucleus (VPM) of the thalamus which reciprocally links the thalamus and the S1BF. VPM was stimulated at 60 v for 0.1 ms.

LFP recordings

Glass electrode micropipettes were filled with an internal solution containing 1 M NaCl with 2% w/v Pontamine sky blue 5BX (BDH Chemicals Ltd., England) and connected to a head-stage amplifier (Cygnus Technology, United States) along with a reference bath Ag/Cl electrode. The signal was relayed to a Neuro Data IR-283 amplifier (10x amplification; Cygnus Technology, United States). Finally, the signal was amplified once more in a custom-built amplifier (100x amplification; Martin Preston, Department of Pharmacology, University of

Oxford, UK) which also performed low-pass filtering with AC/DC input coupling, resulting in a three-step amplification process (totalling a x1000 amplification of original signal) with low-pass filtering at 1000 Hz for final amplified signal. The signal was then transferred to a Micro 1401 mkII AD/DA converter (Cambridge Electronics Design, England). Data were displayed and acquired in Signal software (Cambridge Electronic Design, UK) at 16 kHz.

Experimental sequences

Before imaging commenced, slice stability was confirmed by recording stimulus-evoked LFP responses for 10 min, 15 s ISI. The slice was rejected on the rare occasion that the evoked response deviated by more than 15% in two 10 min sequences. A standard experimental epoch consisted of a 15 min recording period, in which 30 stimuli were presented at 30 s ISI, and then an interval of 10-12 min (Figure S2). If the next epoch included drug administration, the drug perfusate was added at the beginning of this interval, approximately half of the solution in the chamber removed, and perfusion rate increased to 6 mL/min. Drug application was always started with the lowest dose and stability achieved at each dose, before application of the next dose. Stability of slice preparation was confirmed in separate vehicle control experiments were performed with 0.1% DMSO, and showed no modulation of VSDI responses in any brain slices over a 2-hour long recording period (data not shown).

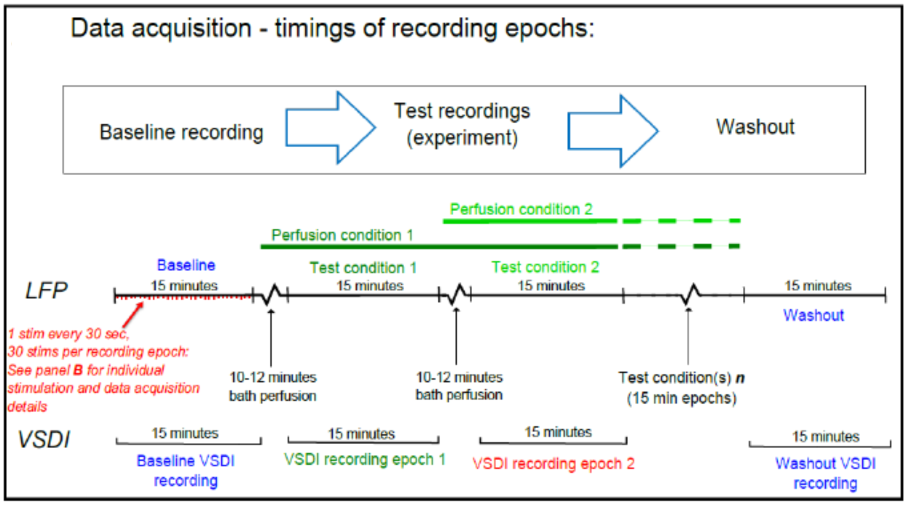

Figure S2. Synchronisation of LFP and VSDI recordings. Having performed a successful stability test, data acquisition proceeds with 15-minute recording epochs interspersed with intervals for washing in any new drug concentration.

Data analysis

MatLab (Mathworks, UK) and Mathematica (Wolfram, United States) were used for data analysis. Individual pixels were expressed as a proportion of baseline fluorescence and a spatial and high-pass temporal filter applied (Figure S3). To allow comparison of responses between slices, a rectangular grid was overlaid upon the region of interest in each brain area (Figure S4). Pixels falling within each rectangle (each 0.12 mm wide) was averaged to generate a two-dimensional activity map (Figure S4, B) and a single medial spatial filter applied. Average responses were produced from each experimental epoch (i.e. average of 30 responses to electrical stimulation of the slice). Three parameters were used: summed fluorescence, lateral spread, and time course of activity. Summed fluorescence was calculated from the average of all segments in an activity map and the sum of fluorescence between 0 and 512 ms above a threshold of 20% of the maximum response. Lateral spread was calculated as the distance between the two points where the response decreased to 20% of the peak response (in mm). The time course was calculated when the response decreased to 20% of the peak response (in ms).

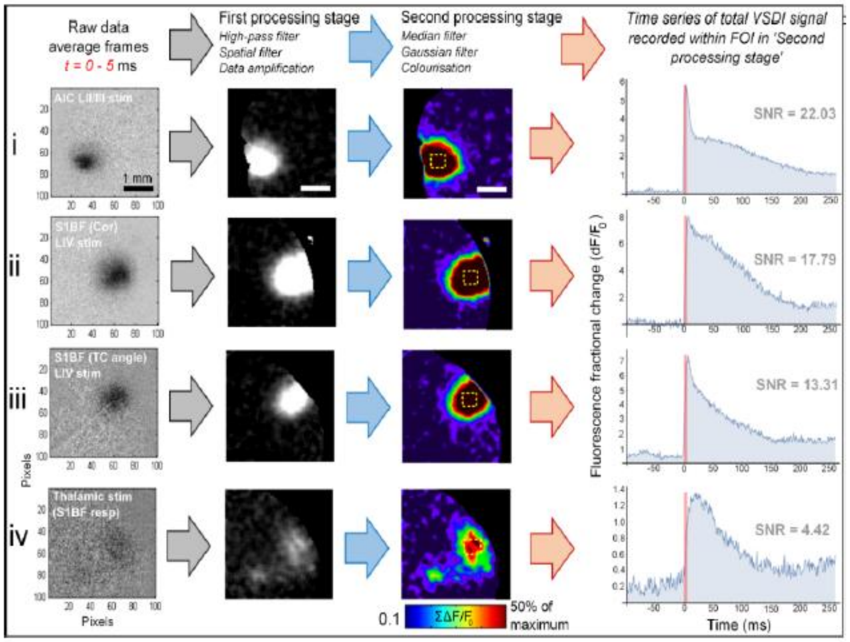

Figure S3. Data analysis. Spatial and high-pass temporal filters were applied to reveal activity maps (central column, 'first processing stage'). For image visualisation only, these data were filtered further with a median and Gaussian filter (right column, 'second processing stage') from which time-series of fluorescence could be produced from selected regions of interest (yellow dotted squares). Processing stages are shown here for assemblies generated in AIC by stimulating layers II/III (i), S1BF by stimulating layer IV (ii), S1BF in the TC slice by stimulating layer IV (iii) and thalamic activation of S1BF (iv). Data show average response to 30 stimulus presentations averaged between 0-5 ms post stimulus (indicated by the red bar in the time-series, right). For visualisation in these example, threshold for max intensity is set at 50% of the response maxima.

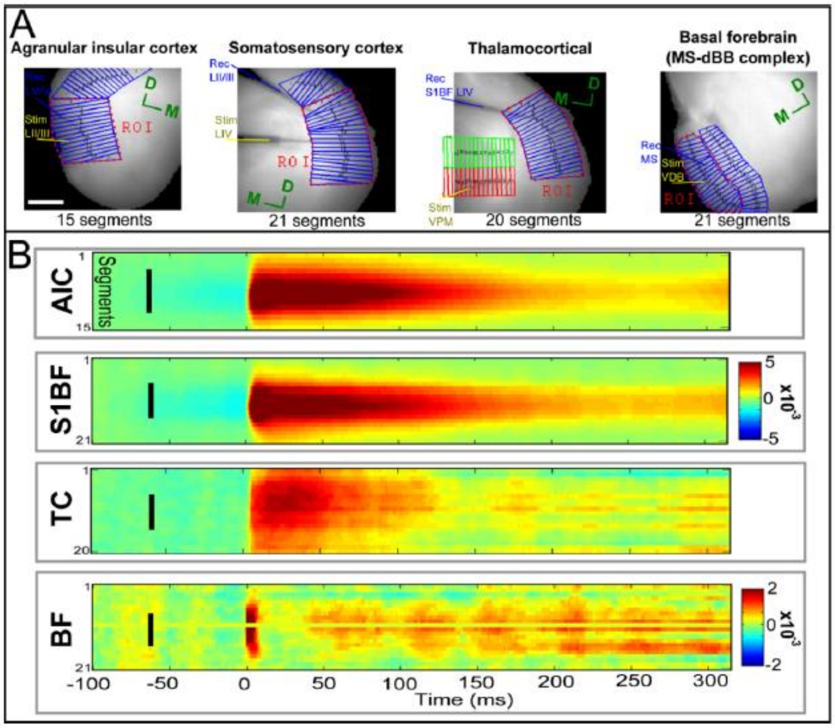

Figure S4. Regions of interest extracted from data. Example data shown are from a baseline recording from AIC, S1BF, TC, and basal forebrain (BF; a region not further described in main manuscript), also known as the mediam septum/diagonal band of Broca (MS/DBB), by stimulation of the vertical limb of the diagonal band. A: grids overlaid in each area (blue lines). B: activity maps generated by averaging each grid rectangle. D = dorsal, M = medial, scale bars = 1 mm.

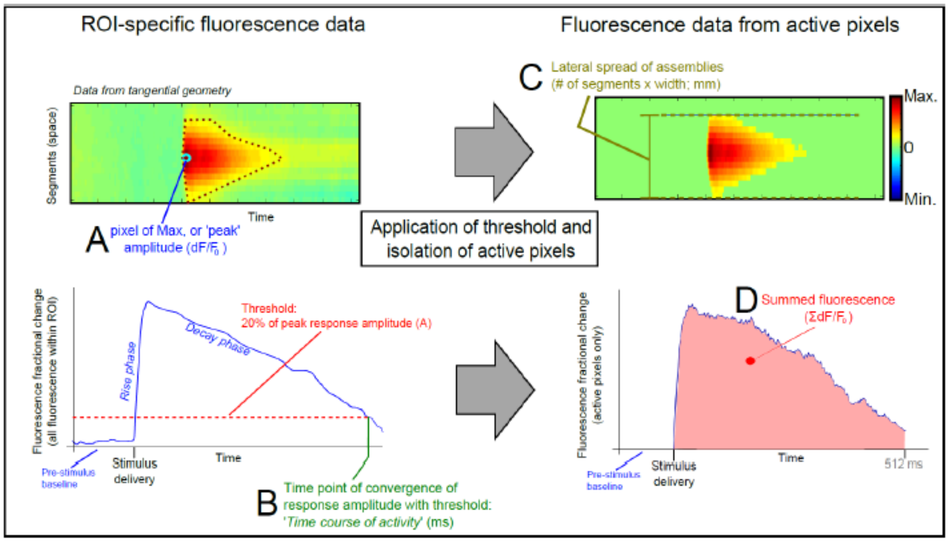

Figure S5. Calculation of imaging parameters. Peak fluorescence is extracted (A) and used to apply a 20% threshold to the data set (bottom-left). The dotted area surrounding the assembly in A represents the shape of the active-pixels (extracted in the top right panel, C). Time course of response is calculated as the point at which activity decreases to 20% of the peak response (B). Active pixels can be visualised in a similar way as the original data, yielding activity maps (top right) and time-series (D) of fluorescence from active pixels only. The lateral spread of activity was calculated (C) by measuring the distance between the points of 20% activity across space. The overall fluorescence was calculated as the area under the curve of pixels above the 20% threshold).

Normalisation of space-time map data

For normalisation of space time maps, the absolute maximum within the overall measured fluorescence from that recording epoch was isolated from its final raster file following which all other values within the matrix data file (i.e. the epoch-average space-time map) were divided by this max amplitude value, producing a space-time map with all values normalised to their absolute maximum (Figure S6).

Using either the original space-time map data produced by the VSDI toolbox or its normalised equivalent, a threshold was applied to the data matrix whereby any data point exhibiting fluorescence values less than 20% of the ‘peak’ maximum fluorescence data point recorded within its respective space-time map had their value converted to 0, while all those above this threshold (active pixels) were converted to 1, producing a binary matrix data set of

active or inactive pixels (Figure S7). Data values of active pixels were then replaced in the binary matrix, producing spacetime maps of active pixels over 20% of max response. This method of applying a minimal threshold for detection and isolation of active pixels clarified the data by removing any background or unspecific activity, and allowed for a much clearer discrimination of evoked responses; such an approach has in fact already been utilised in our previous VSDI investigations.

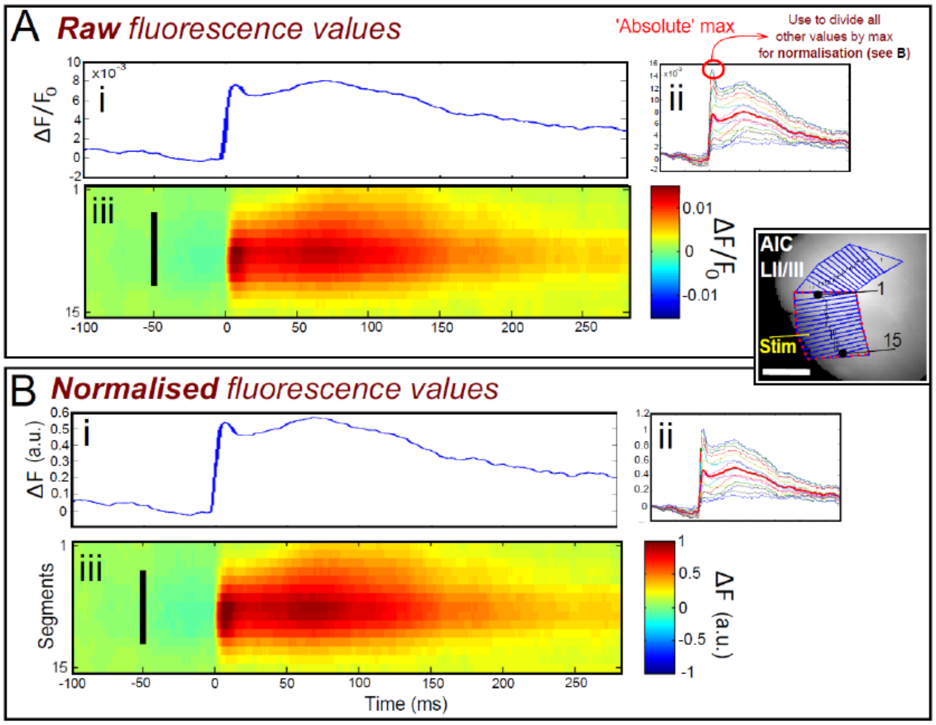

Figure S6. Example data from a single trial showing normalisation of space-time matrix. Stimulation in LII/III of agranular insular cortex (AIC) generated rasterised, 3-dimensional matrices of fluorescence data, i.e. over space and time A, from which the absolute maximum fluorescence reading could be extracted ('Absolute' max, inset time-series graph of individual segment data A) and used to normalise ever other fluorescence (B); black vertical scale bar on space-time maps represents 1 mm in space. Inset shows a bright-field view of the coronal slice containing AIC, the shape of the geometry ROI (dotted red square) used to analyse this specific stimulation paradigm (0.1 ms, 30 V shock), and the arrangement of the segments (blue rectangles). Colour bar represents fluorescence readings, while the (white) scale bar (in slice geometry view, inset) measures 1 mm.

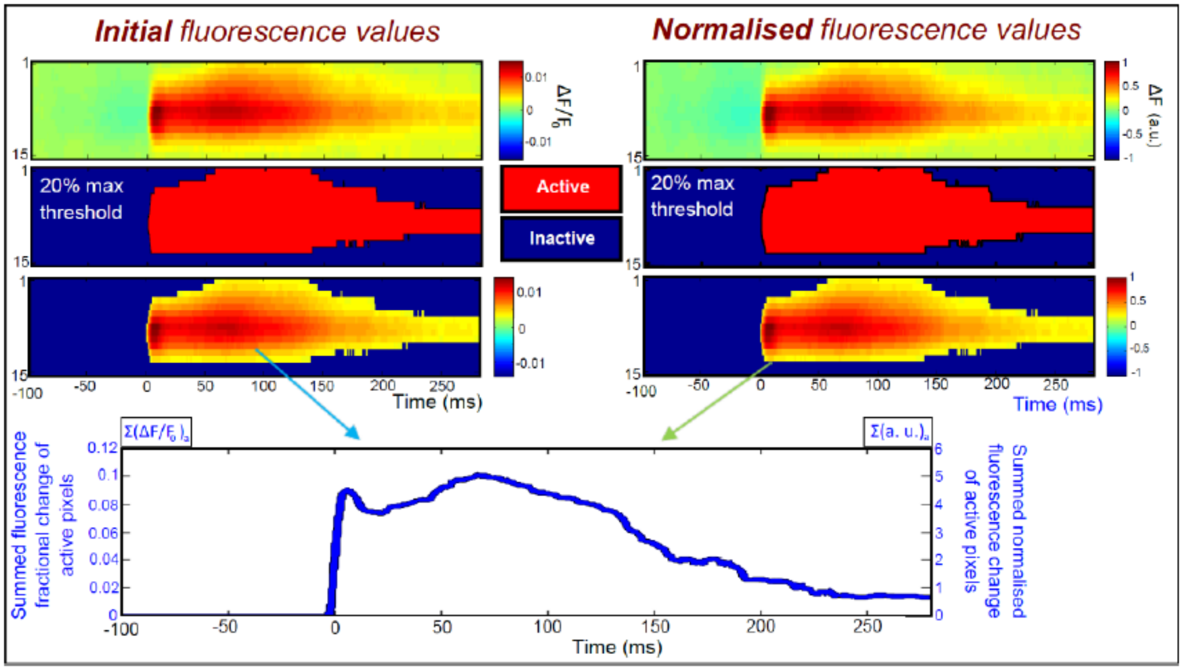

Figure S7. Demonstration of the isolation of active pixels within space-time data sets. With both raw and normalised values, a threshold could be applied which isolated all pixels exhibiting 20% or more of the fluorescence emitted from the absolute maximum amplitude within the data set (middle row). All inactive pixels (below the threshold) had their value turned to 0. When re-colourised (bottom row), the crucial evoked assembly profile of active pixels becomes evident. Finally, in order to quantify the signal originating only from the evoked response (the measurement of interest), the summed fluorescence of active pixels only (Σ(ΔF/F0)a for fluorescence values or Σ(a.u.)a for normalised values) was calculated, yielding the time series of activity shown at the bottom of the figure. Both in the case of raw and normalised data, and once adjusted for axes values, both time-series showed the exact same profile, as can also be seen from their respective space-time map profiles.

Lateral spread was estimated by measuring the largest tangential distance between active pixels (>20% of maximum response) within the region of interest. The time-course of activity was estimated by calculating the time-point at which the time-series activity profiles of individual assemblies crossed the 20% of max threshold (Figure S8).

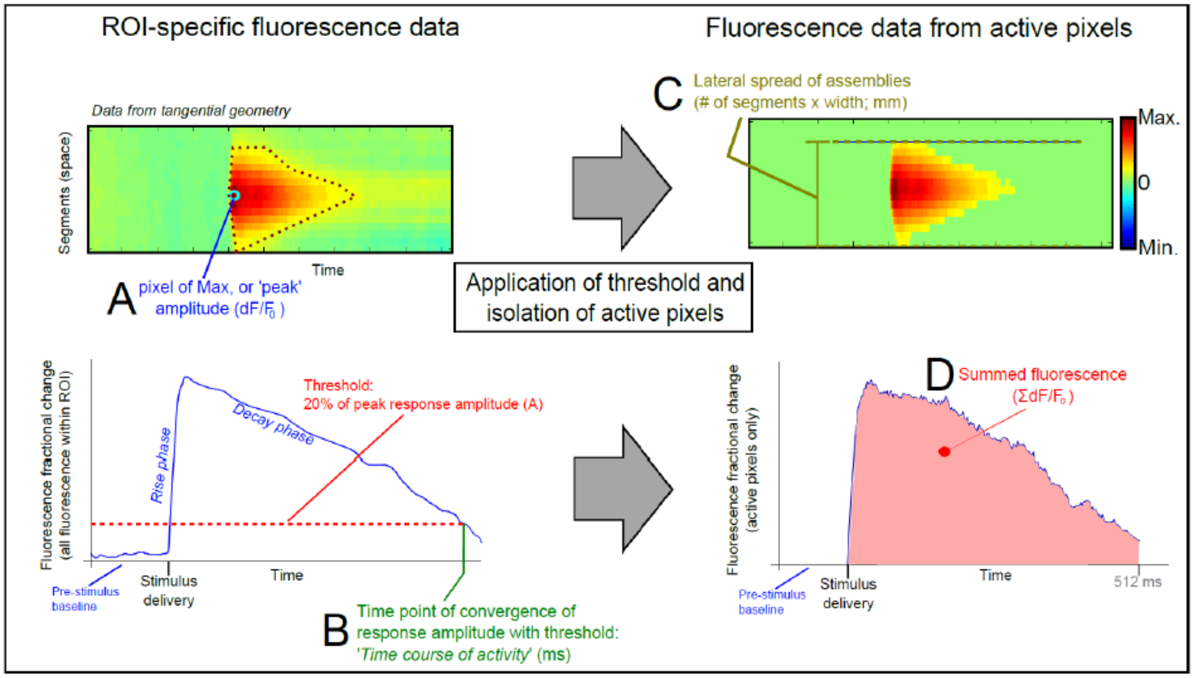

Figure S8. Calculation of quantifiable parameters of assembly dynamics. Initially, data can be visualised either as a space-time map of total fluorescence (top-left), or averaged into a time series (collapsing the spatial dimension, i.e. space-time map y-axis). From the spacetime map, the peak fluorescence reading is isolated (A), and used to apply a 20% threshold of activity (bottom-left) to the data set. The dotted area surrounding the assembly represents the shape of the active-pixels (top right panel), and highlights the level of non-specific data acquired even when taking in account ROI-specific fluorescence (as is the case here, with tangential geometries). The time-point at which the time-series profile of activity crosses the 20% threshold (B) represents the end of the activity time-course of the assembly.

Drug perfusions were inoculated in series starting from a drug-free baseline condition, followed by epochs of successively increasing anesthetic concentrations. In order to prevent the contamination of data as a result of time-dependent changes in assembly dynamics and slice health, experiments were limited to a maximum of 5 successive recording epochs (total of 115-120 minutes). Since modulation of evoked responses over time was inevitable, even over this time frame, a series of control experiments were run with each preparation for that same amount of time and used for normalisation of data. VSDI data were collected as shown in Chapter 3, averaging fluorescence within a region of interest over 15 minute recording periods (30

individual trials), termed ‘recording conditions’ or ‘epochs’. When subsequently running experiments using anesthetics, the data from each recording epoch were normalised in two ways: first, within a single experimental trial, all drug conditions were normalised to their respective baseline, and second: to their respective time-locked control condition, in order to express the data as the percentage change in fluorescence from that which is normally emitted in control experiments. These normalisation steps allowed for the isolation of drug-specific effect, making abstraction of the slowly deteriorating trend in response magnitude which, though non-significant, often become noticeable after 50-60 minutes of recording.

Discussion of threshold and normalisation analysis

Where the process of isolating active pixels presents obvious advantages over the standard averaging of overall fluorescence data sampled within a region of interest, the step of normalisation of active pixel data may present less obvious advantages. However, this normalised version of salient data is in fact very telling: once normalised, time series show the average activity with respect to that data set’s specific fluorescence range - in essence providing a description of the ‘uniformity’ of the evoked response. This data presentation therefore helps characterise key differences in evoked responses between direct and remote cortical activation: that is, thalamocortical activation produced wide-reaching assemblies with low, but more homogenous fluorescence values, whereas direct stimulations produced highly variable fluorescence readings within a comparatively smaller area, i.e. triggering high fluorescence values at the epicentre surrounding the electrode tip and showing a drastic decrease in fluorescence with distance from their epicentre.

Table S1. Statistical comparisons of data in Figures 1.

S1a. Model fits for summed fluorescence. Values are mean (SD); Bmax as normalized units; Kd as µM. ANOVA comparisons are shown across agents (bottom rows) and within each agent across sections (last 2 columns). The agents differ significantly within each section for all pharmacological parameters. Pentobarbital does not exhibit statistically significant differences (at the P<0.05 level) across sections, but ketamine and etomidate do.

	AIC		S1BF		TC		Bmax comparison	Kd comparison
	Bmax	Kd	Bmax	Kd	Bmax	Kd		
Pentobarbital	1.289 (0.344)	758 (329)	1.639 (1.114)	1,361 (1,284)	1.275 (0.147)	284 (74.3)	df 2, F 0.554 p = 0.585	df 2 F 2.976 p = 0.082
Ketamine	0.467 (0.027)	16.8 (4.07)|	0.439 (0.034)	27.9 (9.08)	0.605 (0.036)	14.3 (3.67)	df 2, F 44.69 p <0.001	df 2, F 8.368 p = 0.0036
Etomidate	0.593 (0.0244)	13.5 (2.31)	0.570 (0.016)	13.8 (1.64)	0.782 (0.029)	6.94 (1.23)	df 2, F 143.9 p < 0.001	df 2, F 28.36 p < 0.001
df	2	2	2	2	2	2		
F	29.48	30.58	6.28	6.53	91.40	81.05		
P	<0.001	<0.001	0.0104	0.0091	<0.001	<0.001		

S1b. Goodness of fit for regression lines Figure 1. Regression coefficients (r^2) and mean sum of squares indicate acceptable fits, as indicated by the p-values for the regression.

	Summed fluorescence AIC		p-value for nonlinear regression	Summed fluorescence S1BF		p-value for nonlinear regression	Summed fluorescence TC		p-value for nonlinear regression
	r^2	SS		r^2	SS		r^2	SS	
Pentobarbital	0.966	0.504	0.0004	0.935	0.241	0.0016	0.969	0.441	0.0004
Ketamine	0.918	0.233	<0.0001	0.957	0.186	<0.0001	0.969	0.363	<0.0001
Etomidate	0.991	0.184	0.0004	0.996	0.190	0.001	0.990	0.361	0.0004

Figure S9. Figure 2 in main text re-plotted as concentration-response effects for lateral spread (panels A-C) and time course of activity (panels D-F) for each of the three brain sections: AIC (panels A and D); S1BF (panels B and E) and TC (panels C and F). Plotted values are mean (SEM). Although ligand binding curves could be fit to panels A-C, the extreme sensitivity and immediate saturation of responses with ketamine and etomidate precluded meaningful estimates of Kd or statistical comparison (see supplementary digital content (Table S2c, below). The time course results (D-F) were not subject to statistical analysis but best fit lines were drawn.

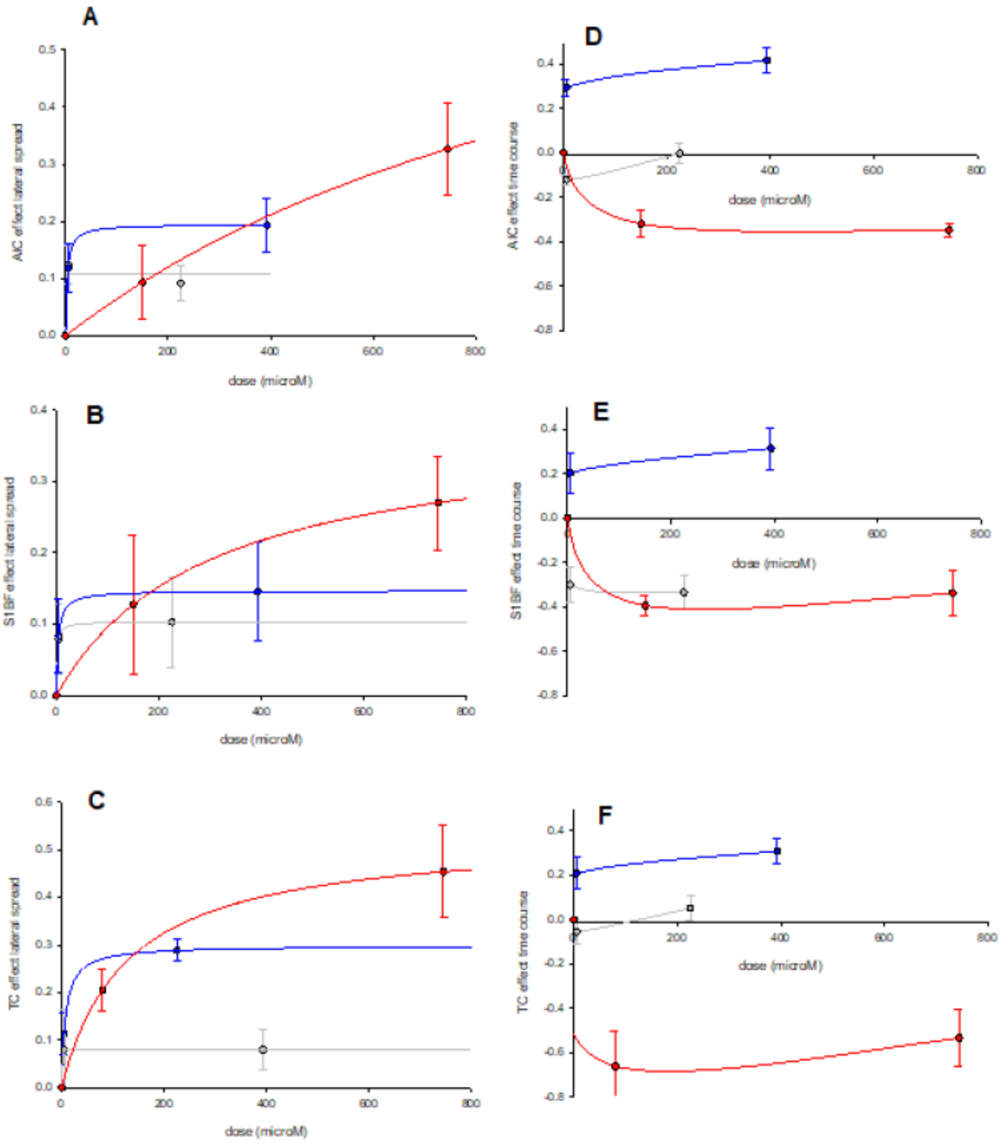

Table S2. Statistical comparisons for lateral spread and time course.

S2a. Attempted model fits for lateral spread in Figure S9. Values are mean (SD); Bmax as normalized units; Kd as μM. The estimates are constrained by the very prompt saturation of response with dose for both ketamine and etomidate.

	AIC		S1BF		TC	
	Bmax	Kd	Bmax	Kd	Bmax	Kd
Pentobarbital	0.884 (0.138)	127 (10)	0.376 (0.106)	294 (75)	0.531 (0.180)	128 (50)
Ketamine	0.195 (0.001)	3.24 (0.001)	0.147 (<0.001)	3.78 (<0.001)	0.299 (<0.001)	8.08 (<0.001)
Etomidate	0.011 (0.024)	<0.001 (1.67)	0.108 (0.024)	<0.001 (<0.001)	0.079 (<0.001)	<0.001 (<0.001)

S2b. ANOVA results for Figure 2 data in main text for lateral spread.

Factors	AIC lateral spread Panel A	S1BF lateral spread Panel B	TC lateral spread Panel C
Drug	0.062	0.443	**0.001**
Dose	**0.017**	0.129	**0.011**
Drug*dose	**0.021**	0.775	0.065
Post hocs			
Etomidate vs ketamine	1.000	1.000	0.197
Etomidate vs pentobarbital	0.062	1.000	0.137
Ketamine vs pentobarbital	0.352	0.679	**<0.001**

S2c. ANOVA results for Figure 2 data in main text for time course.

Factors	AIC time course Panel D	S1BF time course Panel E	TC time course Panel F
Drug	**<0.001**	**<0.001**	**<0.001**
Dose	**0.005**	0.395	0.247
Drug*dose	**0.034**	0.341	0.995
Post hocs			
Etomidate vs ketamine	**<0.001**	**<0.001**	**0.035**
Etomidate vs pentobarbital	**<0.001**	1.000	**<0.001**
Ketamine vs pentobarbital	**<0.001**	**<0.001**	**<0.001**
